# Efficient elimination of pancreatic cancer stem cells by hedgehog/GLI inhibitor GANT61 in combination with mTOR inhibition

**DOI:** 10.1186/s12943-016-0534-2

**Published:** 2016-06-27

**Authors:** Yumi Miyazaki, Shyuichirou Matsubara, Qiang Ding, Koichiro Tsukasa, Makoto Yoshimitsu, Ken-ichiro Kosai, Sonshin Takao

**Affiliations:** Division of Cancer and Regenerative Medicine, Center for Advanced Biomedical Science and Swine Research, Kagoshima University Graduate School of Medical and Dental Sciences, 8-35-1 Sakuragaoka, Kagoshima, 890-8520 Japan; Department of Hematology and Immunology, Kagoshima University Graduate School of Medical and Dental Sciences, Kagoshima University, 8-35-1, Sakuragaoka, Kagoshima, 890-8520 Japan; Department of Gene Therapy and Regenerative Medicine, Kagoshima University Graduate School of Medical and Dental Sciences, Kagoshima University, 8-35-1, Sakuragaoka, Kagoshima, 890-8520 Japan; Tanegashima Medical Center, 7463, Nishi-no-omote, 891-3198 Japan

**Keywords:** Pancreatic cancer, Cancer stem cells, GLI transcription factor, GANT-61, mTOR, Rapamycin

## Abstract

**Background:**

Pancreatic cancer is one of the most lethal malignancies. The innovative treatments are required and now the cancer stem cells (CSCs) are expected to be an effective target for novel therapies. Therefore we investigated the significance of hedgehog (Hh) signaling in the maintenance of CSC-like properties of pancreatic cancer cells, in order to discover the key molecules controlling their unique properties.

**Methods:**

Human pancreatic cancer cell lines, Capan-1, PANC-1, MIA PaCa-2 and Capan-1 M9 were used for our experiments in DMEM/F12 medium containing 10 % fetal bovine serum. Sphere formation assay, immunofluorescence staining, flow cytometric analysis and MTT cell viability assay were performed to investigate molecular signals and the efficacy in the treatment of pancreatic cancer cells.

**Results:**

Inhibition of the Hh pathway significantly reduced the expression of stem cell marker CD133 and sphere formation, an index of self-renewal capacity, demonstrating the suppression of CSC-like properties. Moreover, the GLI inhibitor GANT61 induced greater reduction in sphere formation and cell viability of pancreatic cancer cells than the smoothened (SMO) inhibitor cyclopamine. This suggests that GLI transcription factors, but not SMO membrane protein, are the key molecules in the Hh pathway. The treatment using GANT61 in combination with the inhibition of mTOR, which is another key molecule in pancreatic CSCs, resulted in the efficient reduction of cell viability and sphere formation of an inhibitor-resistant cell line, showing the strong efficacy and wide range applicability to pancreatic CSC-like cells.

**Conclusions:**

Thus, this novel combination treatment could be useful for the control of pancreatic cancer by targeting pancreatic CSCs. This is the first report of the efficient elimination of pancreatic cancer stem-like cells by the double blockage of Hh/GLI and mTOR signaling.

**Electronic supplementary material:**

The online version of this article (doi:10.1186/s12943-016-0534-2) contains supplementary material, which is available to authorized users.

## Background

Pancreatic cancer is one of the most lethal malignancies which the average overall 5-year survival is around 5 % [1]. Therefore, the need for innovative treatments remains urgent. Over the last decade, the cancer stem cell (CSC) hypothesis has developed [2, 3], and is attractive because it may explain the poor prognosis of pancreatic cancer patients.

Pancreatic CSCs have unique functions, including self-renewal, hierarchical proliferation, and “differentiation” into non-self-renewing bulk tumor cells [2, 3]. Further, these CSCs are thought to be correlated with metastasis, chemo- and radio-resistance, and alteration of adjacent stromal cells [4]. Pancreatic CSCs can be distinguished from bulk tumor cells based on their expression of unique surface markers, which include CD133 [2] or a combination of CD44/CD24/EpCAM [3]; their ability to form spheres under non-adherent stem cell culture conditions; and their conclusive ability to form metastases in immunodeficient mice [5].

We recently reported that the mammalian target of rapamycin (mTOR) plays critical roles in maintaining pancreatic CSCs [6], indicating that mTOR may be a promising target to eliminate pancreatic CSCs. In addition, we found that cyclopamine, an inhibitor of the hedgehog (Hh) pathway, significantly reduced the content (percentage) of CD133^+^ cells in a pancreatic cancer cell population. This result indicates that the Hh pathway is another potential target to eliminate pancreatic CSCs. Aberrant expression of the Hh ligand is observed at a high frequency in pancreatic cancer and is detectable throughout disease progression [7] because pancreatic CSCs have been reported to express elevated level of the Hh ligand [3].

Activation of the canonical Hh signaling pathway is initiated by the binding of Hh ligands, such as sonic hedgehog (SHH), to the transmembrane receptor patched (PTC). This activates another transmembrane signaling molecule smoothened (SMO). Subsequently SMO activates the final mediator of Hh signaling, the GLI family of transcription factors. The activation of GLI family results in the expression of Hh target genes [7]. Blockage of Hh signaling has been examined to prevent disease progression and metastatic spread using predominantly Hh/SMO signaling (i.e., Hh signaling at the level of the SMO transmembrane molecule) inhibitors. However, these inhibitors were not so effective for many cancers in which Hh ligand overexpression is considered to drive tumor growth [8]. The efficacy of the Hh/SMO signaling inhibitors on pancreatic cancer is still in dispute.

A small molecule inhibitor of GLI1 and GLI2, the *G*li *ANT*agonist (GANT61), was recently identified. This molecule acts in the nucleus to block GLI1- and GLI2-mediated transcription, and shows a high specificity for Hh signaling [9]. We applied this molecule to treat pancreatic CSC-like cells and found that targeting Hh/GLI signaling (i.e., Hh signaling at the level of the GLI transcription regulator) effectively reduces CSC-like properties. Based on these results, we investigated the efficacy of combination treatment of GANT61 and the mTOR inhibitor (rapamycin) to pancreatic cancer cell lines. This is the first report indicating the effective and powerful elimination of pancreatic CSCs by blockage of both Hh/GLI and mTOR signaling.

## Methods

### Reagents

We purchased antibodies against SHH (rat mAb) and Notch1 (rabbit mAb) from Abcam (Cambridge, UK), antibodies against GLI1 (mouse mAb) from Santa Cruz Biotechnology, Inc. (Santa Cruz, CA, USA), antibodies against β-catenin (mouse mAb) from BD Transduction Laboratories (Lexington, KY, USA), antibodies against CD133 (mouse mAb clone AC133) and CD44 (mouse mAb clone DB105) from Miltenyi Biotec (Cologne, Germany), respectively. We obtained. GANT61 (2,2′-[[Dihydro-2-(4-pyridinyl)-1,3(2H,4H)-pyrimidinediyl]bis(methylene)]bis(N,N-dimethylbenzenamine)), cyclopamine, and rapamycinfrom Sigma-Aldrich (St. Louis, MO, USA), and SAG (3-chloro-N-[(1r,4r)-4-(methylamino)cyclohexyl]-N-[3-(pyridin-4-yl)benzyl]benzo[b]thiophene-2-carboxamide) from Merck-Calbiochem (Darmstadt, Germany).

### Cell cultures

Human pancreatic cancer cell lines, Capan-1, PANC-1 and MIA PaCa-2 were purchased from the American Type Culture Collection (ATCC, Manassas, VA, USA) and cultured in DMEM/F12 medium containing 10 % fetal bovine serum (Invitrogen, Carlsbad, CA, USA), at 37 °C under a humidified atmosphere of 95 % air and 5 % CO_2_. We established the Capan-1 M9 cells as described previously [10].

### Sphere formation assay

#### Multicellular sphere formation assay

Single-cell suspensions were plated in a 24 well ultra-low attachment surface culture plate (Corning, NY, USA) at a density of 1000 cells per well in 0.5 ml medium and other conditions are same as the previously described methods for single cell sphere formation [6].

#### Single cell sphere formation assay

Single-cell suspensions were plated in a 96 well ultra-low attachment surface culture plate (Corning, NY, USA) by limiting dilution method at a density of 0.7 cells per well in 0.1 ml medium and other conditions are same as multicellular sphere formation assay above.

### Immunofluorescence staining

We fixed the cells in 4 % paraformaldehyde for 10 min at room temperature followed by permeabilization with 0.2 % Triton X-100 in PBS and blocking with 2 mg/ml BSA, then incubated them with primary antibodies overnight at 4 °C. Afterwards, we incubated the cells with fluorescent-labeled secondary antibodies.

### Flow cytometric analysis

Cells were stained with the mouse anti-human CD133 mAb (allophycocyanin (APC)-conjugated) and the mouse anti-human CD44 mAb (R-phycoerythrin (PE)-conjugated) and analyzed with a FACSAria II flow cytometer (Becton Dickinson, Franklin Lake, NJ, USA) [5].

### MTT cell viability assay

2 × 10^3^ cells were seeded in a 96-well plate and treated with the inhibitors for three days. Then the cell viability was determined by MTT assay [5].

### siRNA transfection and Real-time quantitative PCR (RT-PCR)

GLI1-siRNA was purchased from ambion (Life Technologies, CA, USA) and transfected using Lipofectamine™ RNAiMAX transfection reagent according to the manufacturer’s instructions.

Briefly, single cell suspension containing 2 × 10^5^ cells in 2 ml of DMEM/F12 with 10 % FCS were mixed with RNAi duplex consisting of 50 pmol siRNA and 3.5 μl RNAiMAX reagent and plated in a well of 6 well plate (first/reverse transfection). Cells were cultured for 48 h, and then the medium was changed with DMEM/F12 with 10 % FCS containing 50 pmol siRNA and 3.5 μl RNAiMAX reagent (second/forward transfection). Incubation was continued for 48 h and cells were used for experiments.

Total RNA was extracted using TRIzol (Invitrogen, CA, USA) and Direct-zol RNA MiniPrep Kit (Zymo Research, CA, USA) according to the manufacturer’s instructions. For messenger RNA quantification, primers, probes and 2× Taqman Universal PCR Master Mix were obtained from Applied Biosystems^TM^ (Life Technologies, CA, USA). RT-PCR was performed in accordance with the manufacturer’s instructions, using a StepOne Real-time PCR system (AppliedBiosystems, CA, USA). Glyceraldehyde-3-phosphate dehydrogenase (GAPDH) was used for normalization.

### Two dimensional culture colony formation assay

Single cell suspension was prepared and cells were seeded into100 μl of DMEM/F12 containing 10 % FCS in single wells on 96-well plates using a FACS Aria II system. Seven days after seeding, the number of wells containing colonies were measured.

### DNA-microarray analysis

Total RNA was extracted using an RNeasy extraction kit (Qiagen, Hilden, Germany). The cDNA was amplified, labeled, and hybridized to a 44 K Agilent 60-mer oligo microarray according to the manufacturer’s instructions. All hybridized microarray slides were then scanned by an Agilent scanner. Relative hybridization intensities and background hybridization values were calculated using Agilent Feature Extraction Software.

### In vivo treatment of xenograft with GANT61 and rapamycin

The animal study was approved by the Committee on the Use of Live Animals for Teaching and Research of Kagoshima University. BALB/c nu/nu (nude) mice were purchased from CLEA Japan (Tokyo, Japan). For in vivo treatment of GANT61 and/or rapamycin, nude mice were randomly assigned to four groups at two weeks after s.c. injection of Capan-1 M9 cells (5 x10^5^ cells per injection). Mice received treatments of 40 mg/kg/day GANT61 (i.p.), 1 mg/kg/day rapamycin (i.p.), or their vehicle (20 % ethanol in corn oil for GANT61and 5 % PEG400, 5 % Tween-80 and 4 % ethanol for rapamycin, i.p.) 3 times a week for 25 days. The tumors were measured after a 4 or 5-day interval; the tumor volume was calculated as follows: tumor volume = length × width^2^/2.

### Statistical analyses

The results are expressed as the mean ± standard deviation (s.d.). We evaluated comparisons between the averages of two groups using a two-tailed Student’s *t*-test. We considered *P* < 0.05 to be statistically significant.

## Results

### Hedgehog signaling components are expressed in pancreatic cancer cells

**Fig. 1:**
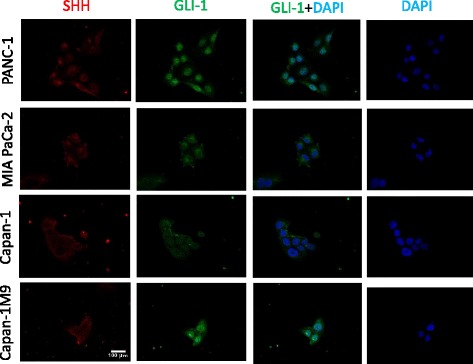
Immunofluorescence detection of the ligand and mediator of hedgehog (Hh) signaling in a culture of pancreatic cancer cells. GLI1: green, sonic hedgehog (SHH): red, DAPI: blue. Scale bar: 100 μm. The experiment was repeated three times and the representative results were indicated. Negative controls, the staining only with secondary antibodies, are depicted in Additional file 1: Figure S1A

In order to confirm the expression of Hh signaling components, we performed immunofluorescence staining of pancreatic cancer cell lines with anti-SHH or anti-GLI1 antibodies. Three typical cell lines of pancreatic cancer (PANC 1, MIA PaCa-2, and Capan-1) and one subline established in our laboratory (Capan-1 M9 [10], see 3.2) showed positive staining with anti-SHH and anti-GLI1 antibodies (Fig. 1). It is important that GLI1 staining revealed the nuclear dominant pattern, because GLI1/2, which is the final mediator of the pathway, accumulates in the nucleus and activates the transcription of target genes upon the activation of Hh signaling [7]. In contrast, we could not confirm the nuclear staining of β-catenin or Notch (cytoplasmic tail) (Additional file 1: Figure S1B), although frequent activation of these signals in pancreatic cancer and their crucial functions in CSCs are reported [7]. These molecules act as the mediators for Wnt/β-catenin or Notch signaling by similar mechanisms, i.e., nuclear translocation and target gene activation. No staining of β-catenin nor Notch in the nucleus may correlate with the ineffectiveness of the inhibitors for Wnt/β-catenin and Notch signaling pathways on pancreatic cancer cells [6] (Additional file 1: Figure S2).

### Signaling in the Hedgehog pathway regulates pancreatic cancer stem cell characteristics

To evaluate the significance of Hh signaling in the maintenance of CSC properties, we examined the effect of Hh signaling inhibitors on pancreatic cancer cell line Capan-1 M9. This subline is a highly migratory and invasive subline selected from the human pancreatic cancer cell line, Capan-1, and displays an elevated expression of CD133 (approximately 90 % of the cells express CD133) [10] (also see Additional file 1: Figure S3). We used this subline as an in vitro CSC model system for CD133^+^ pancreatic cancer stem-like cells, because CD133^+^ Capan-1 cells were identified as a population of cancer stem-like cells [6, 11].

**Fig. 2:**
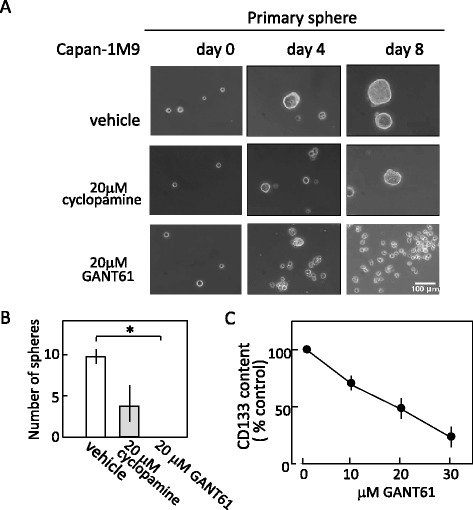
Inhibition of hedgehog (Hh) signaling reduced the self-renewal as evidenced by sphere formation and the expression of stem cell marker CD133 of pancreatic cancer cells. **a** Representative photographs of Capan-1 M9 spheres cultured in a stem cell culture medium in the absence or presence of 20 μM of inhibitors, cyclopamine or GANT61. Scale bar: 100 μm. **b** The effects of cyclopamine and GANT61 on the number of spheres. Primary spheres were cultured in triplicate and the sphere numbers that were larger than 100 μm in diameter were counted per well. The results are presented as the mean and s.d. of triplicates obtained in 1 representative experiment out of 2.*P < 0.05. **c** The effects of GANT61 on the frequency of CD133^+^ cells. The results are presented as percentages of control values in untreated cells, showing the mean and s.d. of triplicates obtained in 1 representative experiment out of 2

First, we examined the effect of Hh inhibitors on the sphere formation (multicellular sphere formation assay) of Capan-1 M9 cells. Spheroid formation is an index of the cells’ self-renewal capacity, which is a major property of stem cells [12]. Cancer stem-like cells have been cultured under bFGF (+) EGF (+) and serum-free conditions to investigate their sphere-forming ability [2, 13]. Under stem cell growth conditions, Capan-1 M9 cells formed spheres that reached 140 μm in diameter after 8 days (Fig. 2a). Treatment with inhibitors, cyclopamine and GANT 61 at 20 μM, significantly reduced both the size and number of spheres formed. Spheres that were larger than 100 μm in diameter are depicted in Fig. 2b. Cyclopamine reduced the sphere number by half: in contrast, GANT61 actually abolished the formation of spheres of this size. These results indicate that Hh signaling is essential for the self-renewal of CD133^+^ pancreatic cancer cells as exhibited by the sphere formation and the Hh/GLI inhibitor, GANT61, reduced more efficiently than the Hh/SMO inhibitor, cyclopamine.

Our previous study [6] (Additional file 1: Figure S2) and others [14] have reported that the Hh/SMO inhibitor cyclopamine reduces the CD133 expression in pancreatic cancer cells. To examine whether or not the Hh/GLI inhibitor GANT61 also reduces CD133 expression, we treated Capan-1 M9 cells with GANT61 for 3 days and then analyzed them by FACS. Treatment with GANT61 reduced the amount of CD133^+^ cells in the Capan-1 M9 cells in a concentration-dependent manner (Fig. 2c and Additional file 1: Figure S3), highlighting that the reduction of CD133 expression is a common feature of Hh inhibitors. The significance of Hh/GLI signaling to maintain CSC properties was also supported by the analysis of stem cell markers. Again, GANT61 induced greater reduction in the CD133 content (percentage) of pancreatic cancer cells than cyclopamine (compare Fig. 2c with Additional file 1: Figure S2).

Collectively, these results demonstrate that Hh signaling plays a critical role to maintain CSC-like properties of pancreatic cancer cells. Furthermore, the Hh/GLI inhibitor, GANT61, was more efficient in blocking the function of these cells than the Hh/SMO inhibitor, cyclopamine.

### CD133^+^ pancreatic cancer cells are sensitive to hedgehog/GLI inhibitor GANT 61

**Fig. 3:**
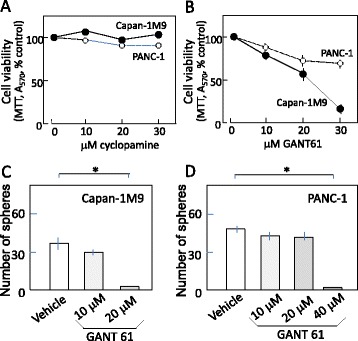
The hedgehog (Hh)/GLI inhibitor GANT61 reduced the viability and sphere formation of pancreatic cancer cells; PANC-1 is a less sensitive cell line. **a**, **b** The hedgehog (Hh)/GLI inhibitor GANT61, but not the Hh/SMO inhibitor cyclopamine, significantly reduced the viability of pancreatic cancer cells. The results are presented as percentages of control values in untreated cells, showing the mean and standard deviation (s.d.) of 4 replicates obtained in 1 representative experiment out of 3. Closed circle: Capan-1 M9. Open circle: PANC-1. **c** GANT61 reduced the sphere formation of Capan-1 M9 cells in a single cell sphere formation assay. We determined the number of spheres formed per 96-well plate in triplicate. All data are mean and s.d. in 1 representative experiment out of 2. **d** GANT61 reduced the sphere formation of PANC-1 cells at a higher concentration. The results are presented as (**c**). *P < 0.05

It has been reported that Hh signaling is blocked more efficiently by GANT61 at the level of GLI1/GLI2, which in turn induces DNA damage and cell death in human colon carcinoma cells, compared with the SMO inhibitor cyclopamine [15]. The effects of GANT61 on the cell viability of CD133^+^ pancreatic cells was examined. We treated Capan-1 M9 cells with either cyclopamine or GANT61 for 3 days prior to performing an MTT cell viability assay. The cell viability of Capan-1 M9 cells was reduced by GANT61 in a concentration-dependent manner (Fig. 3b), whereas cyclopamine did not have any detectable effect on viability (Fig. 3a). Thus, targeting GLI1/GLI2 (GANT61) would induce a greater effect on CD133^+^ cell viability than targeting SMO (cyclopamine).

### The SMO-agonist SAG does not stimulate the sphere formation

To evaluate the impact of SMO stimulation on sphere formation of CD133^+^ cells, we apply the SMO-agonist SAG to Capan-1 M9 cells. The treatment of Capan-1 M9 cells with 20 μM GANT61 in the single cell sphere formation assay actually abolished the formation of spheres (Fig. 3c), showing a similar result observed in multicellular sphere formation assay (see Fig. 2b). In contrast, the treatment of Capan-1 M9 cells with 1 nM – 1 μM SAG does not affect the sphere formation significantly (Additional file 1: Figure S4). This may indicate that Hh/SMO signaling is less important to maintain pancreatic CSC-like properties or the signaling is saturated without stimulation by SAG.

### PANC-1 cells are resistant to GANT61 compared to CD133^+^ pancreatic cancer cells

On the other hand, we examined the effect of GANT61 on another pancreatic cancer cell line PANC-1, which is reported as a cyclopamine resistant cell line [16]. Although we have not identified the cancer stem cell marker for PANC-1 cells, PANC-1 has been shown to form tumors in immunodeficient mice (Additional file 1: Table S1), indicating that it contains the cells with CSC-like properties. The effects on cell viability of PANC-1 cells were much modest than those on Capan-1 M9 cells, though the reduction of viability in PANC-1 cells was still statistically significant at 30 μM by GANT61 (*P* < 0.01, Fig. 3b).

To analyze the effects of GANT61 on the sphere formation of PANC-1 cells, we cultured PANC-1 cells in 96-well plates using the limiting dilution method (single cell sphere formation assay) [12]. When PANC-1 cells are plated in 24 wells with 1000 cells per well, which is the plating condition in multicellular sphere formation assay, they form a large aggregate (Additional file 1: Figure S5A). Thus the determination of sphere-forming ability by the multicellular method is not possible for PANC-1 cells. Under stem cell culture conditions, PANC-1 cells formed spheres of 200 μm in diameter after 8 days (Additional file 1: Figure S5B). The administration of 20 μM GANT61 did not reduce the number of spheres (Fig. 3d). A higher concentration of GANT61 (40 μM) is required to inhibit the sphere formation of PANC-1 cells (Fig. 3d). Therefore, it is suggested that the cancer stem-like cells in PANC-1 show much greater resistance to GANT61 than those in the Capan-1 M9 cells in a sphere formation assay.

### GLI1 knockdown reduces the sphere formation and cell viability of CD133^+^ pancreatic cancer cells

**Fig. 4:**
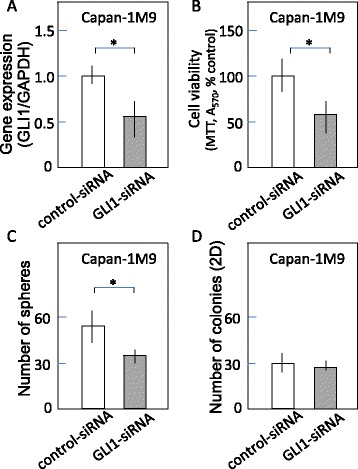
GLI1 knockdown reduces the cell viability and sphere formation of pancreatic cancer cells but not the colony formation in two dimensinal culture. **a** GLI1 expression after GLI1-si RNA transfection measured by RT-PCR. **b** Reduction of cell viability by GLI1-siRNA. Cells were transfected for 96 h, then followed by MTT assay. The results are presented as percentages of control values in untreated cells, showing the mean and s.d. of 4 replicates obtained in 1 representative experiment out of 2. **c** Inhibition of sphere formation by GLI1-siRNA. Cells were transfected for 96 h, then followed by single cell sphere formation assay. The results are presented as in Fig. 3c. **d** Effect of GLI1-siRNA on the colony formation in two-dimensional culture. Cells were transfected for 96 h, then single-cell suspensions were plated in 96 well plate as 1 cell/well. After 7 days incubation, the number of colonies formed in a plate were counted. The results are presented as the mean and s.d. of 3 replicates obtained in 1 representative experiment out of 2. *P < 0.05

To confirm that GLI is indeed the important and key molecules to maintain CSC-like properties of pancreatic cancer cells, we knocked down GLI1 in Capan-1 M9 cells. GLI2 expression was not detectd in this cell line by RT-PCR (data no shown). The transfection of siRNA against GLI1 significantly reduced the mRNA level (Fig. 4a). GLI1 knockdown reduces the sphere formation and cell viability of Capan-1 M9 cells (Fig. 4b and c). In contrast, the colony formation in 2 dimensional culture was not reduced by the transfection of GLI-siRNA (Fig. 4d). These results support the notion that that GLI is the key molecules in the Hh pathway to maintain CSC-like properties of pancreatic cancer cells, and exclude GANT61-mediated off target or simply toxic effects in the sphere formation assay.

In addition, DNA-microarray analysis revealed that GLI1 is upregulated in spheres (Additional file 1: Figure S6). This also supports the significant function of GLI in sphere formation.

### Combination treatment with the hedgehog/GLI inhibitor and the mTOR inhibitor

We demonstrated that Hh/GLI signaling plays a critical role in maintaining the CSC-like properties and the viability of pancreatic cancer cells. Hence, targeting the pathway by GANT61 is a potentially effective method to treat the pancreatic CSCs. To provide a robust therapeutic approach against the resistant cell population such as PANC 1 cells, we tried the combination treatment of pancreatic cancer cells with the mTOR inhibitor rapamycin. A single treatment of 20 μM GANT61 or 100 nM rapamycin did not reduce the number of spheres in PANC-1 cells. In contrast, the combination of these two inhibitors strongly reduced the sphere formation of PANC-1 cells (Fig. 4a). These results provide the proof that double blockage of Hh/GLI and mTOR signaling strongly suppress the self-renewal of cancer stem-like cells which show a low sensitivity to GANT61.

In addition, this combination improved the effect of inhibitors on the viability of the PANC-1 cells. After a single treatment with either 40 μM GANT61 or 100 nM rapamycin, 40–50 % of the cells remained viable. Combination treatment with the two inhibitors reduced cell viability to approximately 15 % (Fig. 4b). When we added a low concentration of gemcitabine, a conventional anticancer drug, to this combination, viable cells virtually disappeared, whereas the same concentration of gemcitabine alone only reduced the cell viability to approximately 50 %.

We confirmed the effect of combination treatment of GANT61 and rapamycin also in Capan1M9 cells (Additional file 1: Figure S7A). In addition, in vivo treatment of the combination of GANT61 and rapamycin on xenograft tumors derived from this cell line suppressed tumor growth (Additional file 1: Figure S7B).

The combination treatment with the Hh/GLI inhibitor GANT61 and the mTOR inhibitor rapamycin strongly reduced the sphere formation and cell viability of pancreatic cancer cells, suggesting the possibility of controlling pancreatic CSCs by blocking both these signaling pathways.

## Discussion

The discovery and development of CSC-targeting therapies are dependent on the identification of key molecules controlling the unique properties of CSC populations. In this study, we investigated the significance of Hh signaling for the maintenance of CSC-like properties using typical cell lines of pancreatic cancer. We found that the inhibitors of Hh signaling reduced self-renewal ability, as indicated by sphere formation and the expression of a CSC marker CD133, demonstrating the crucial function of the Hh pathway in pancreatic CSCs. Furthermore, we demonstrated that the Hh/GLI inhibitor GANT61 is more efficient in reducing the CSC-related properties than the Hh/SMO inhibitor cyclopamine, indicating GLI as a key molecule in the pathway.

A spheroid formation is a major property of CSCs under serum-free culture conditions, and used frequently to show the self-renewal ability of CSCs in vitro. CSC marker is another method to recognize CSCs, and important in clinical analysis. The up-regulation of CD133, a marker of CSCs, in pancreatic cancer correlates with poor prognosis of patients [17]. There was a significant correlation between CD133 expression and the tumorigenicity, chemoresistance, and migration and invasion ability of Capan-1 cells [10]. After the treatment with gemcitabine, CD133^+^ cell content (percent) is increased in Capan-1 cells. This may be caused by a higher capacity of CD133^+^ cells to resist the anticancer drag for the standard therapy. In contrast, the inhibitors of Hh signaling reduce the content of CD133^+^ cells. GANT61 showed 80 % reduction at high concentration while the reduction by cyclopamine reached plateau around 50 %. In addition, the cell viability of Capan-1 M9 cells was reduced linearly by GANT61, whereas cyclopamine did not have any detectable effect. Therefore, the viability of CD133^+^ cells, which would be indicated as a multiplication of CD133^+^ cell content and the total cell viability including both CD133^+^ and CD133^−^ cells, is reduced much more efficiently by GANT61 than cyclopamine. Not only in sphere formation but also in the analysis of CSC marker CD133, the Hh/GLI inhibitor in blocking the critical function of Hh signaling in CSC-like cells was more efficient than the Hh/SMO inhibitor.

Multistage development of pancreatic cancer in mouse models is not affected by the deletion of the SMO, which is essential for the transduction of the canonical Hh signal from the extracellular ligand [18]. According to the report, autocrine SHH-Ptch-SMO signaling is not required for cancer progression and alternative mechanisms keeping the expression of GLI target genes exist in pancreatic cancer cells. Those mechanisms, namely the noncanonical activation pathway of GLI, may underlie the ineffectiveness of Hh/SMO inhibitors. Hh/SMO inhibitors cannot block the noncanonical Hh signal. It is important that disappointing results have been reported in the clinical trials of Hh/SMO inhibitors for pancreatic cancer [7]. Collectively, the key molecules controlling the properties of pancreatic CSCs in the Hh pathway are GLI transcription factors, and the correlation between SMO and CSC-like properties is rather restricted.

On the basis of the above notion that GLI transcription factors are the key molecule in the Hh pathway, we proposed a novel combination treatment on pancreatic CSCs with the Hh/GLI inhibitor GANT61 and the mTOR inhibitor rapamycin. Targeting the Hh pathway by GANT61 is a potentially effective method, however the sensitivity to GANT 61 varies among cell lines, suggesting that some pancreatic cancer cell populations may show the resistance to GANT61 in a manner similar to PANC-1 cells. GLI and mTOR are two key molecules in the different signaling pathways, therefore, will show synergistic effect. As expected, the combination treatment effectively reduced the sphere formation and cell viability of PANC-1, the inhibitor-resistant cell line. This novel combination would improve the efficacy and the applicability of therapeutic treatments, much more than GANT61 single treatment [19] or the combination treatment of rapamycin with Hh/SMO inhibitors [13, 20].

**Fig. 5:**
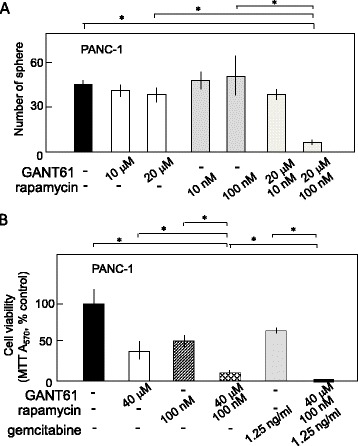
**a** The hedgehog (Hh)/GLI inhibitor GANT61 (20 μM) in combination with the mTOR inhibitor rapamycin (100 nM) effectively reduced the sphere formation of PANC-1 cells. The results are presented as in Fig. 3c. **b** The hedgehog (Hh)/GLI inhibitor GANT61 (40 μM) in combination with the mTOR inhibitor rapamycin (100 nM) effectively reduced the viability of PANC-1 cells. The results are presented as in Fig. 3 a and b. *P < 0.05

Several studies have reported the molecular interaction of Hh and mTOR signaling, suggesting possible mechanisms of synergistic effects. Recently the crosstalk between the PI3K/Akt/mTOR and Hh pathways, which is mediated by S6 kinase (S6K), was described in esophageal adenocarcinoma [21] and in PTEN-deficient glioblastoma [22]. S6 kinase S6K1 is activated downstream of the PI3K/Akt/mTOR pathway and phosphorylates GLI1, resulting in SMO-independent noncanonical GLI1 activation. In these tumors, simultaneous inhibition of the Hh pathway by an Hh/SMO inhibitor, and mTOR/S6K signaling by PI3K and/or mTOR inhibitor showed enhanced efficacy by avoiding the redundancy of canonical and noncanonical GLI activation (Fig. 5). On the other hand, induction of apoptosis by the treatment of GANT61 and PI103, a PI3K/mTOR inhibitor, shows synergistic effect but the signals are mediated through different effector molecules in rhabdomyosarcoma. GANT61 single treatment upregulates the proapoptotic protein NOXA while PI103 single treatment upregulates another proapoptotic protein BMF [23]. This finding cannot be explained only by the crosstalk between S6K and GLI, suggesting existence of other mechanism. In our experiments, Hh and mTOR inhibitors showed different effect on CD133 expression (Fig. 2c and Additional file 1: Figure S2). This result also suggests the existence of mTOR specific function, not via GLI activation by S6K (Additional file 1: Figure S1). Further analysis will clarify the precise mechanism of synergistic effect in pancreatic CSCs.

## Conclusions

We investigated the significance of Hh/GLI signaling in pancreatic CSCs to maintain their stemness-related properties. On the basis of the identification of this key molecule, we proposed and demonstrated a novel combination treatment using the Hh/GLI inhibitor GANT61 with mTOR inhibition. This combination will provide an efficient approach to control CSCs in pancreatic cancer and will improve the therapeutic options for this devastating disease.

## Abbreviations

CSCs, cancer stem cells; Hh, hedgehog; Hh/GLI signaling, hedgehog signaling at the level of the GLI transcription regulator; Hh/SMO signaling, hedgehog signaling at the level of the smoothened transmembrane molecule; mTOR, mammalian target of rapamycin; PDAC, pancreatic ductal adenocarcinoma; PTC, patched; SHH, sonic hedgehog; SMO, smoothened.

## Additional file

Additional file 1: Figure S1. Staining of Capan-1 M9 cells without first antibodies as negative controls (A) and with antibody against β-catenin or Notch 1 (intra cellular domain: NICD) (B). Scale bar: 100 μm. Similar results were obtained in the staining of PANC-1, MIA PaCa-2 and Capan-1 cells (data not shown). The experiment was repeated three times and the representative results were indicated. **Figure S2.** The effects of signaling inhibitors on the frequency of CD133^+^ cells and the viability of pancreatic cancer cells. We treated Capan-1 M9 cells with the inhibitors at the indicated concentration for 3 days, then determined the frequency of CD133^+^ cells and their cell viability by flow cytometry and the MTT assay, respectively. The results are presented as percentages of control values in untreated cells, showing the mean and standard deviation (s.d.) of 4 replicates obtained in 1 representative experiment. **Figure S3.** The effects of GANT61 on the surface markers and the shape of Capan-1 M9 pancreatic cancer cells. (A) Flow cytometric analysis of CD133 and CD44 expression on Capan-1 M9 cells treated with or without GANT61. We treated the Capan-1 M9 cells for 3 days, then analyzed them. (B) Representative photographs of Capan-1 M9 cells untreated and treated with GANT61. GANT61 treatment induced a morphologic change of the Capan-1 M9 cells. This may correlate with the increased baseline level in the flow cytometric analysis of GANT61 treated cells shown in (A), which reflect the changes in the structure of the cell surface. Scale bar: 100 μm. **Figure S4.** The SMO-agonist SAG does not stimulate the sphere formation in Capam1M9 cells. We treated Capan-1 M9 cells with indicate4d concentration of SAG during sphere formation and determined the number of spheres formed per 96-well plate in triplicate. All data are mean and s.d. in 1 representative experiment out of 2. **Table S1.**Tumorigenesis of pancreatic　cancer cell lines in NOD/SCID mice. **Figure S5.** The limiting dilution method is necessary to analyze the sphere-forming ability of PANC-1 cells. (A) PANC-1 cells form large aggregates in the multicellular sphere formation assay. We plated single-cell suspensions (10^3^ cells in 0.5 ml) in 24 wells and cultured them. Representative photographs from day 2. (B) Representative photographs of PANC-1 spheres in the single-cell sphere formation assay. We plated single-cell suspensions (1.2 cells in 100 μl) in 96 wells and cultured them for 8 days. Scale bar: 100 μm. **Figure S6.** A heatmap of the DNA microarray demonstrates upregulation of GLI1 expression in spheres of Capan-1 M9 cells under stem cell culture condition. M9: Capan-1 M9 cells in two-dimensional culture with 10 % FCS, M9 Sphere: Capan-1 M9 spheres formed in a stem cell culture medium. **Figure S7.** (A) The hedgehog (Hh)/GLI inhibitor GANT61 (10 μM) in combination with the mTOR inhibitor rapamycin (10 nM) effectively reduced the sphere formation of Capan-1 M9 cells. The results are presented as in Fig. 3c. (B) The hedgehog (Hh)/GLI inhibitor GANT61 in combination with the mTOR inhibitor rapamycin impairs in vivo growth of xenografted pancreatic carcinoma in nude mice. Tumor growth of Capan-1 M9 xenografts which were treated with vehicle, 40 mg/kg of body weight, 1 mg/kg of body weight, or combination. Tumor volume was shown as the mean and SD from 5 mice (10 tumors) from each group. * *P* < 0.05. **Figure S8.** A schematic representation of the possible mechanism(s) underlying the synergistic effect of double blockage of Hh and mTOR signaling. The decoupling of GLI activation from SHH-PTCH-SMO signaling and/or the redundancy of canonical and noncanonical GLI activation may underlie the ineffectiveness of Hh/SMO inhibitors. This makes GLI as the key molecule in Hh pathway. The difference in the effect of Hh/GLI inhibitors (which reduces CD133^+^ cell content) and the mTOR inhibitor (which does not reduce CD133^+^ cell content) suggests that the mTOR function is not mediated only by S6K. Assuming the specific function of mTOR (*) will explain the different effect. (PDF 466 kb)
