# Supplementary material for: Efficient elimination of pancreatic cancer stem cells by hedgehog/GLI inhibitor GANT61 in combination with mTOR inhibition
Source: Mol Cancer. 2016 Jun 27;15:49. doi: 10.1186/s12943-016-0534-2 (PMC4924257; doi:10.1186/s12943-016-0534-2)
Supplement: Additional file 1: Figure S1. — Staining of Capan-1 M9 cells without first antibodies as negative controls (A) and with antibody against β-catenin or Notch 1 (intra cellular domain: NICD) (B). Scale bar: 100 μm. Similar results were obtained in the staining of PANC-1, MIA PaCa-2 and Capan-1 cells (data not shown). The experiment was repeated three times and the representative results were indicated. Figure S2. The effects of signaling inhibitors on the frequency of CD133+ cells and the viability of pancreatic cancer cells. We treated Capan-1 M9 cells with the inhibitors at the indicated concentration for 3 days, then determined the frequency of CD133+ cells and their cell viability by flow cytometry and the MTT assay, respectively. The results are presented as percentages of control values in untreated cells, showing the mean and standard deviation (s.d.) of 4 replicates obtained in 1 representative experiment. Figure S3. The effects of GANT61 on the surface markers and the shape of Capan-1 M9 pancreatic cancer cells. (A) Flow cytometric analysis of CD133 and CD44 expression on Capan-1 M9 cells treated with or without GANT61. We treated the Capan-1 M9 cells for 3 days, then analyzed them. (B) Representative photographs of Capan-1 M9 cells untreated and treated with GANT61. GANT61 treatment induced a morphologic change of the Capan-1 M9 cells. This may correlate with the increased baseline level in the flow cytometric analysis of GANT61 treated cells shown in (A), which reflect the changes in the structure of the cell surface. Scale bar: 100 μm. Figure S4. The SMO-agonist SAG does not stimulate the sphere formation in Capam1M9 cells. We treated Capan-1 M9 cells with indicate4d concentration of SAG during sphere formation and determined the number of spheres formed per 96-well plate in triplicate. All data are mean and s.d. in 1 representative experiment out of 2. Table S1.Tumorigenesis of pancreatic cancer cell lines in NOD/SCID mice. Figure S5. The limiting dilution method is necessary to analyze [file 12943_2016_534_MOESM1_ESM.pdf]

Additional file for Y. Miyazaki et al.

Efficient elimination of pancreatic cancer stem cells by Hedgehog/GLI inhibitor GANT61 in combination with mTOR inhibition

Additional file 1: Table S1

Tumorigenesis of pancreatic cancer cell lines in NOD/SCID mice.

Cell line	Number of cells for implantation	tumorigenesis
Capan-1 CD133^{+}	1000	5/5
Capan-1 CD133^{--}	1000	0/5
PANC-1 (unsorted cells)	1000	3/3

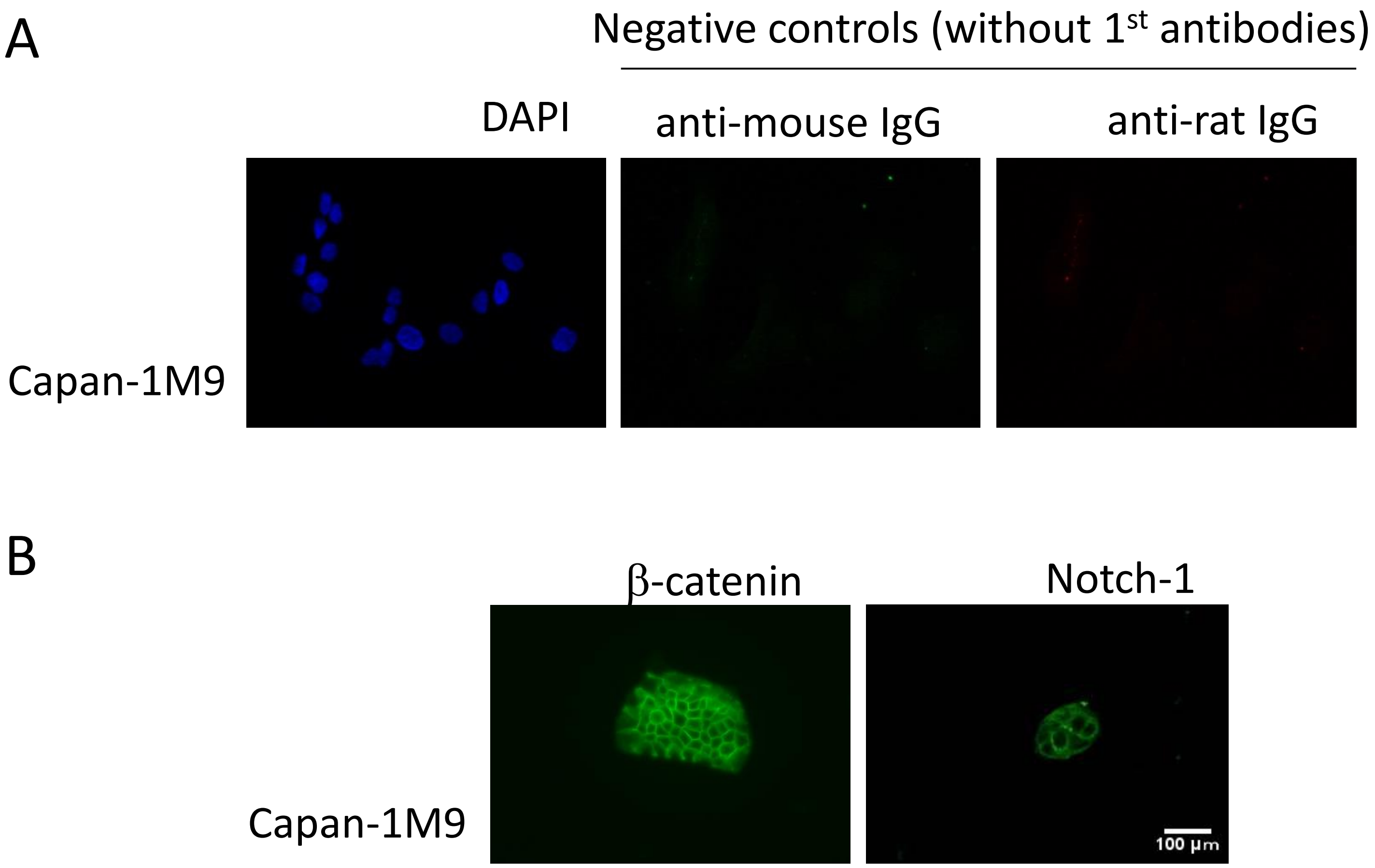

Additional file 1: Figure S1

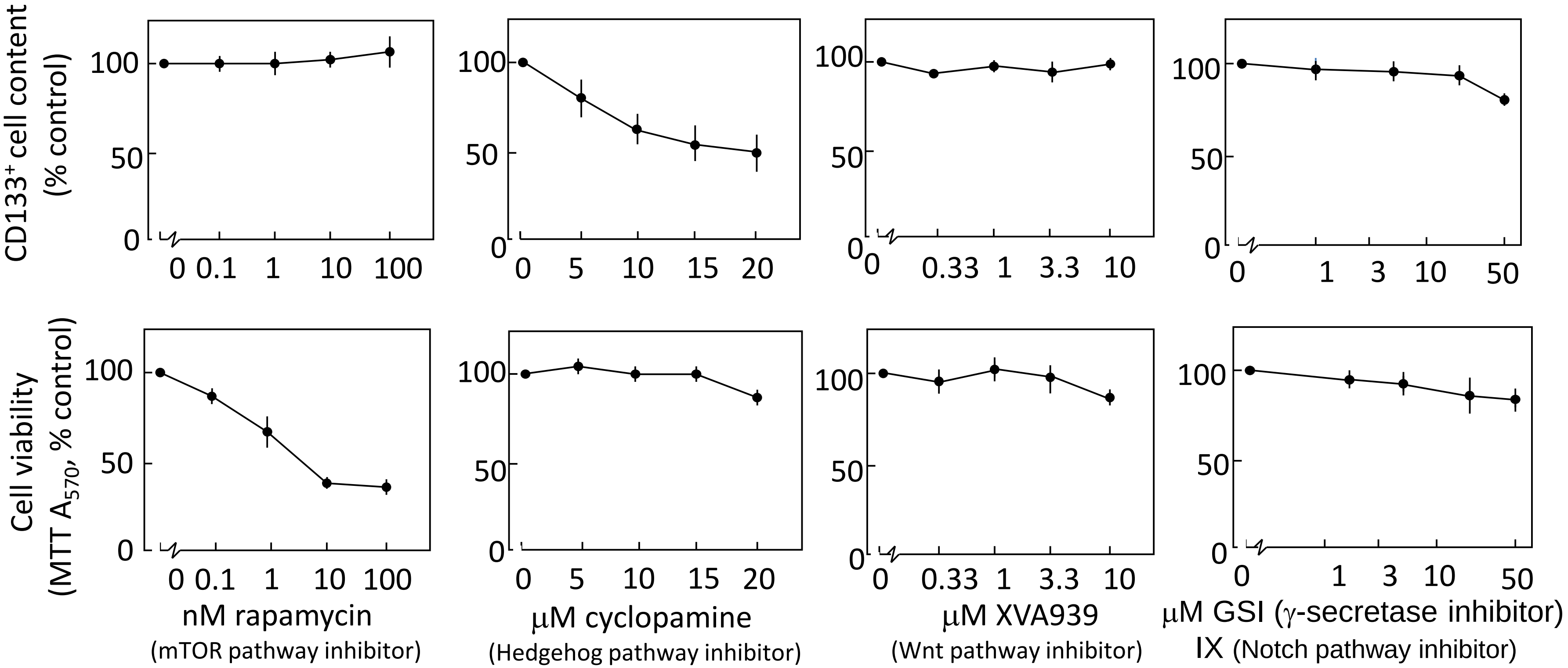

Additional file 1: Figure S2

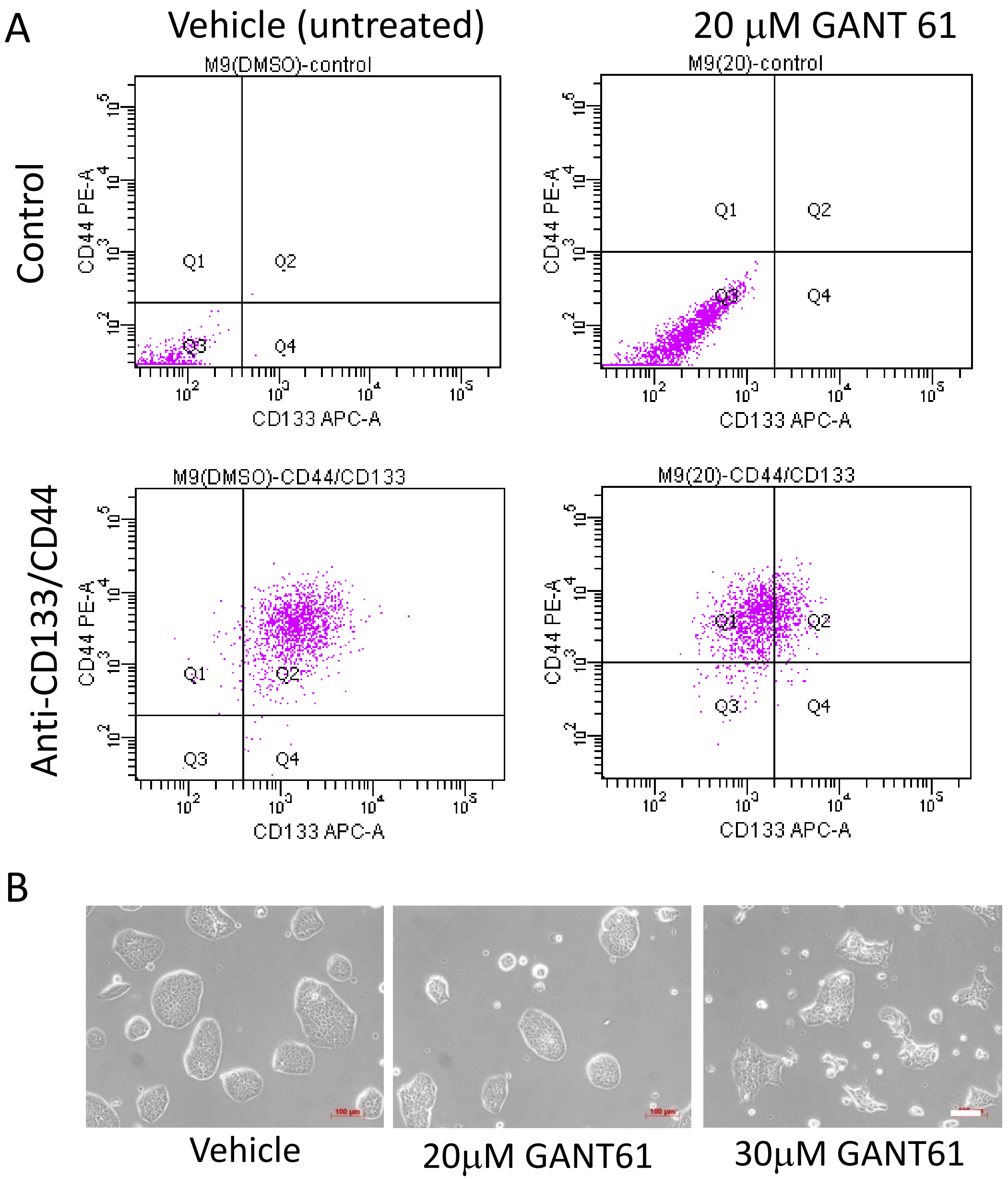

Additional file 1: Figure S3

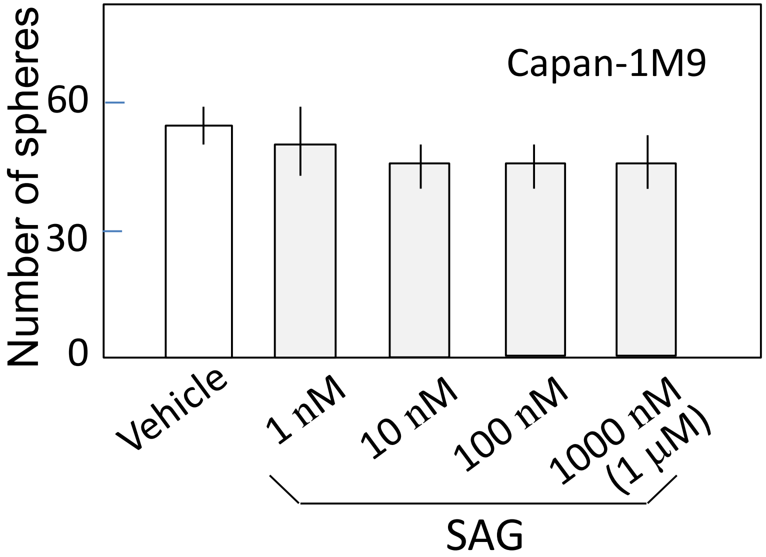

Additional file 1: Figure S4

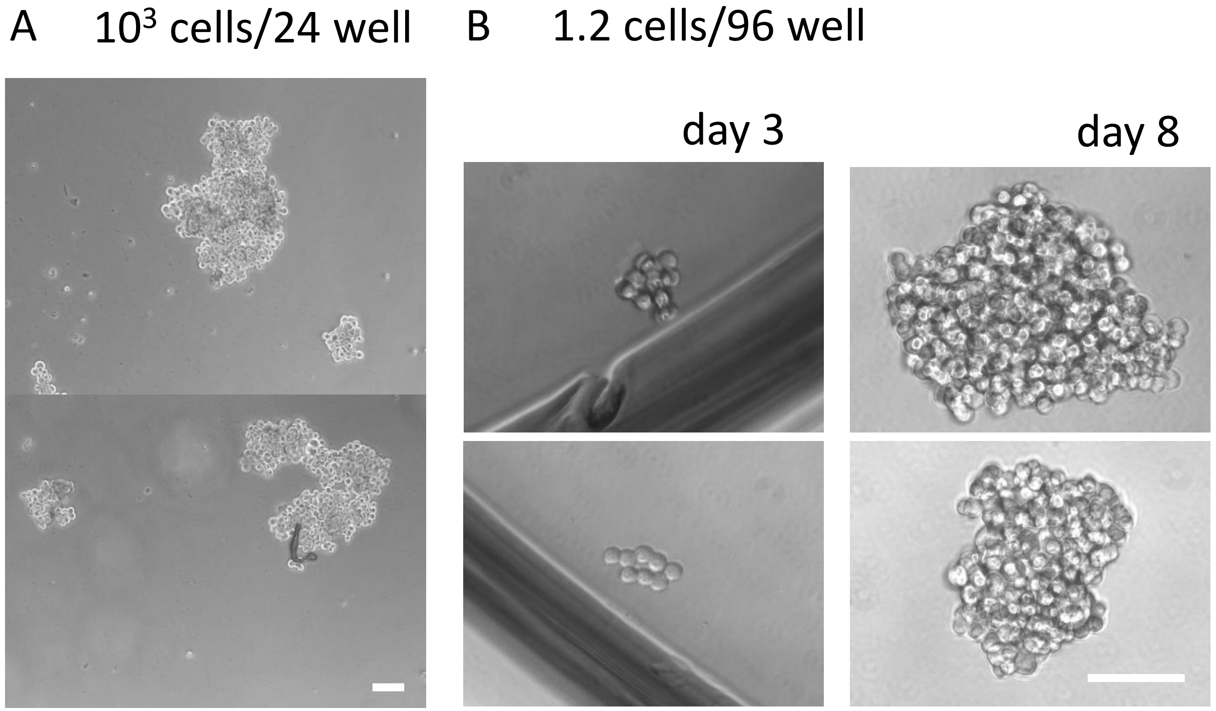

Additional file 1: Figure S5

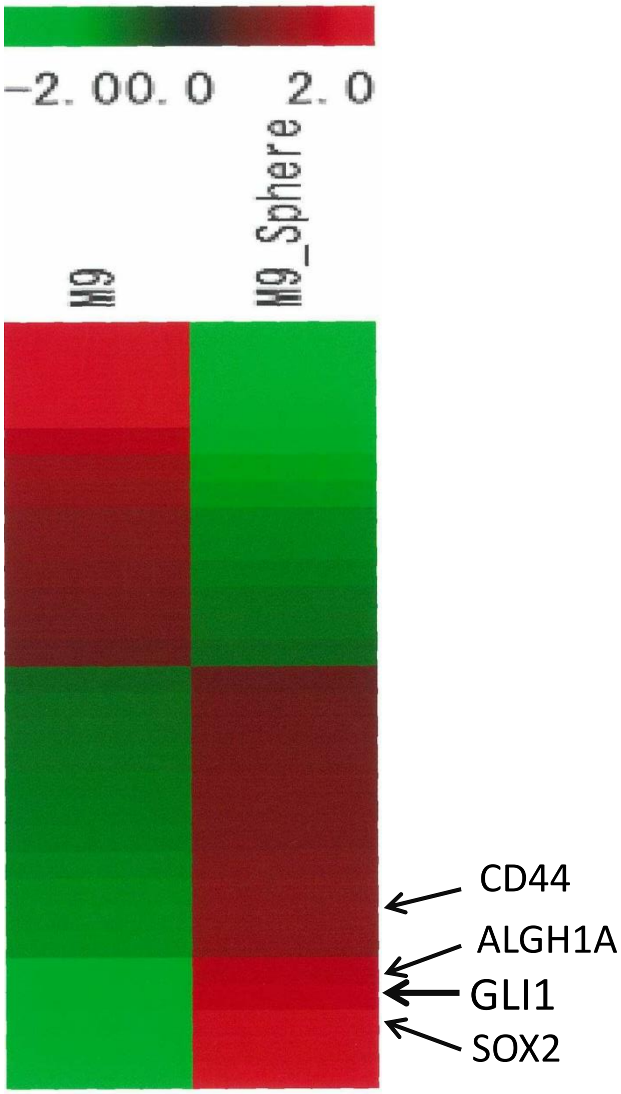

Additional file 1: Figure S6

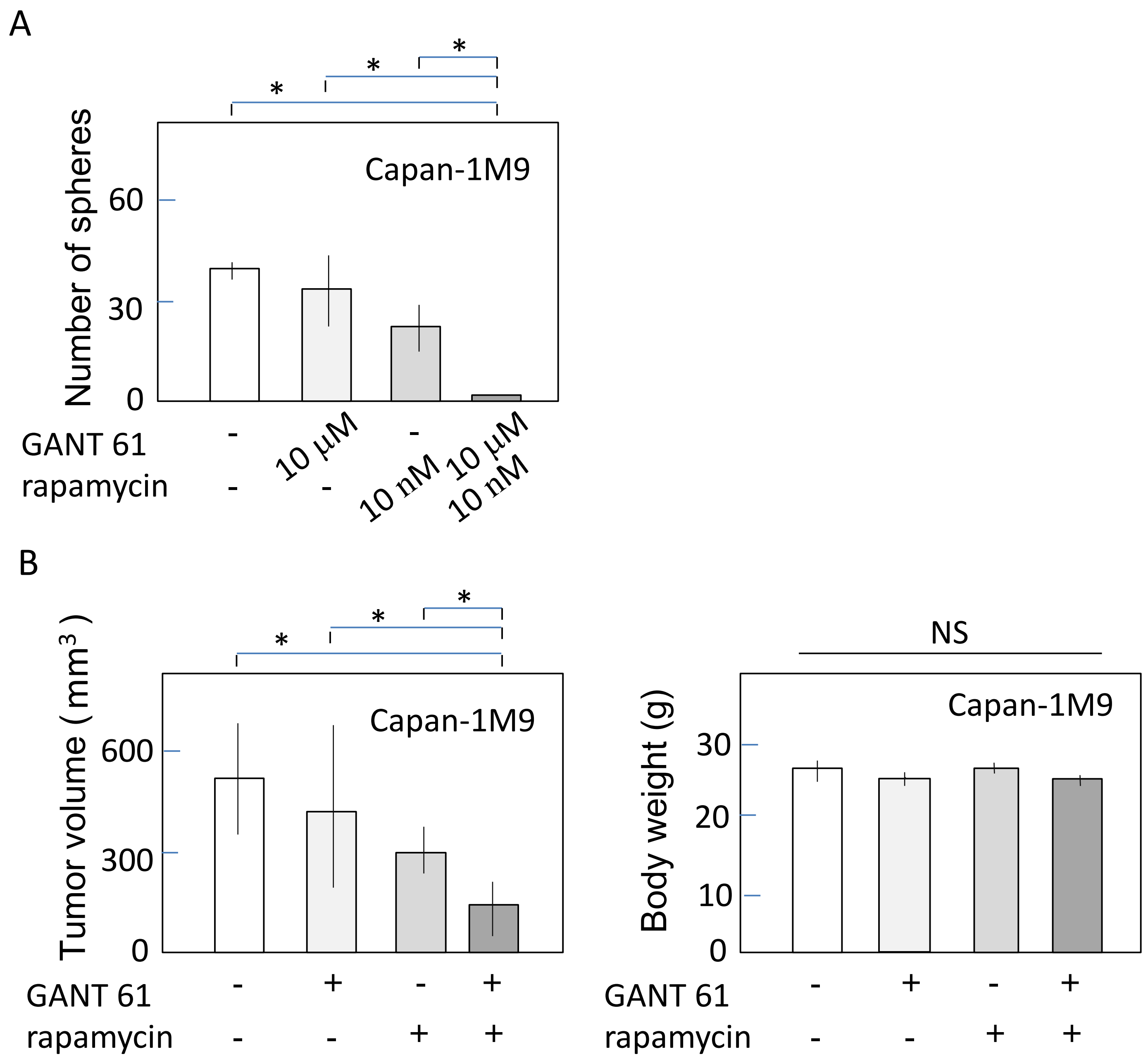

Additional file 1: Figure S7

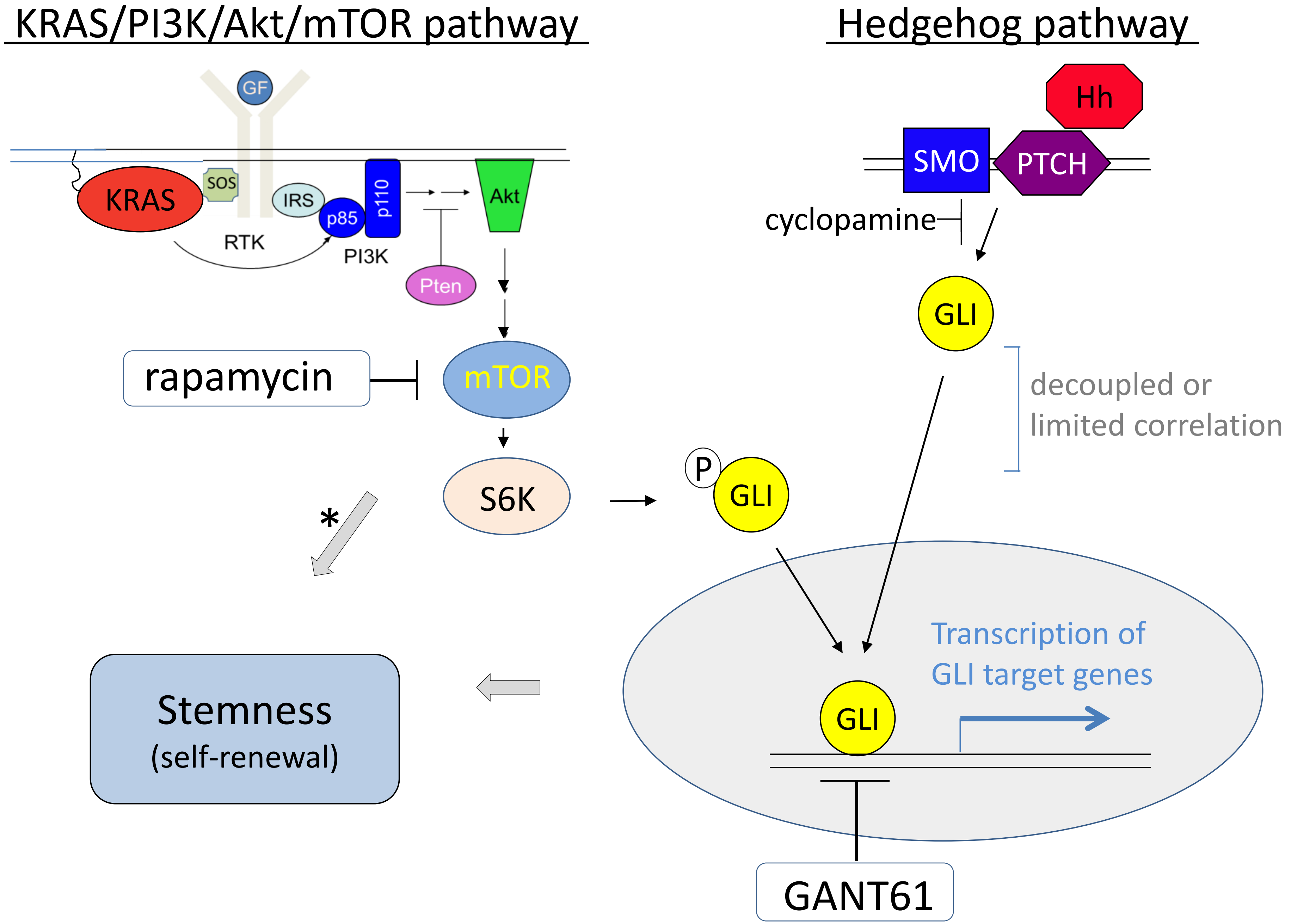

Additional file 1: Figure S8
